# Reliability of Pseudotyped Influenza Viral Particles in Neutralizing Antibody Detection

**DOI:** 10.1371/journal.pone.0113629

**Published:** 2014-12-01

**Authors:** Jinghui Yang, Weidong Li, Yunfeng Long, Shaohui Song, Jing Liu, Xinwen Zhang, Xiaoguang Wang, Shude Jiang, Guoyang Liao

**Affiliations:** 1 Institute of Medical Biology, Chinese Academy of Medical Sciences & Peking Union Medical College, Kunming, Yunnan, China; 2 Minhang District Center for Disease Control and Prevention, Shanghai, China; Johns Hopkins University - Bloomberg School of Public Health, United States of America

## Abstract

**Background:**

Current influenza control strategies require an active surveillance system. Pseudotyped viral particles (pp) together with the evaluation of pre-existing immunity in a population might satisfy this requirement. However, the reliability of using pp in neutralizing antibody (nAb) detection are undefined.

**Methodology/Principal Findings:**

Pseudotyped particles of A(H1N1)pmd09 (A/California/7/2009) and HPAI H5N1 (A/Anhui/1/2005), as well as their reassortants, were generated. The reliability of using these pp in nAb detection were compared concurrently with the corresponding viruses by a hemagglutination inhibition test, as well as ELISA-, cytopathic effect-, and fluorescence-based microneutralization assays. In the qualitative detection on nAbs, the pp and their corresponding viruses were in complete agreement, with an R^2^ value equal to or near 1 in two different populations. In the quantitative detection on nAbs, although the geometric mean titers (95% confidence interval) differed between the pp and viruses, no significant difference was observed. Furthermore, humoral immunity against the reassortants was evaluated; our results indicated strong consistency between the nAbs against reassortant pp and those against naïve pp harboring the same hemagglutinin.

**Conclusion/Significance:**

The pp displayed high reliability in influenza virus nAb detection. The use of reassortant pp is a safe and convenient strategy for characterizing emerging influenza viruses and surveying the disease burden.

## Introduction

Influenza viruses have caused flu pandemics multiple times throughout history. There have been four major flu pandemics since 1918. The 1918–1919 pandemic H1N1 virus infected approximately 20–40% of the world's population and led to an estimated death toll of 50 million people, while the 1957–1958 pandemic H2N2 virus originated in Asia and led to 1–1.5 million deaths. Similarly, the 1968–1969 pandemic H3N2 virus killed an estimated 1 million people worldwide. Most recently, the 2009 pandemic H1N1 influenza virus resulted in an estimated 151,700–575,400 deaths worldwide during its first year of circulation [1]. New influenza viruses emerge constantly. For example, a novel avian influenza A virus strain, H7N9, raised considerable concern worldwide in 2013, while the highly pathogenic avian influenza (HPAI) H5N1 virus has circulated in Europe and Asia for more than a decade and has spread to more than 60 countries; thus far, it has infected 650 humans and killed 386 of them [2]. Although reports of human-to-human HPAI H5N1 transmission are rare [3], [4], its high lethality has raised significant concern worldwide.

Along with advancements in biomedical technology and collaboration among international organizations and national governments, the responses to previous communicable disease pandemics have resulted in the following standard procedures: disease surveillance, pathogen identification, epidemic situational reporting and surveillance, public health interventions when necessary, and vaccine and drug development [5]–[7]. Although these procedures have improved disease control and prevention worldwide, they are generally passive defenses. Many additional procedures should be considered, including origin studies of novel pathogens, background data collection for particular infectious diseases, pandemic trend and pandemic scale surveillance, accuracy assessments of the disease burden, and examinations of regional disparity. These complementary approaches would promote an active surveillance system and prevent unnecessary social panic and financial loss.

Pre-existing immunity is an important factor that affects pandemic trends and limits the pandemic scale of communicable diseases [8], [9]. Regarding the 2009 influenza pandemic, despite the initially high mortality rate in Mexico, the virus caused generally mild symptoms and the overall mortality was around 0.45% [10]; this is not significantly higher than that of seasonal influenza [11]. Pre-existing immunity has been assumed to contribute to the overall low morbidity of the 2009 pandemic H1N1 virus [9], [12]. Evidence has shown that the spectra of pathogens vary geographically [13]; thus, the patterns of pre-existing immunity to a certain pathogen and pandemic scale also differ. An inapparent infection with a certain virus or infection with related viruses could evoke immune protection in subsequent contacts. This immune protection varies regionally because inapparent infections are probably dependent on the actual distribution of a certain pathogen; local human, livestock, and poultry population densities; environmental quality; and the sociocultural history. For example, in mainland China, the prevalence of antibodies against hepatitis A and E viruses in individuals over the age of 40 years is approximately 100 and 40%, respectively, which is significantly higher than in Western countries. Thus, there is an established immunologic barrier among Chinese adults against hepatitis A and E viruses [14], [15]. Therefore, studies of the background immunity in a population will greatly benefit the scientific and rational response to a pandemic. Pandemic trend, pandemic scale, and disease burden surveillance mechanisms could be set up by virus reassortment predictions and the evaluation of background immunity in a population.

Current major approaches to the evaluation of humoral immunity include the hemagglutination inhibition test and ELISA-, cytopathic effect (CPE)-, and fluorescence-based microneutralization assays. However, these methods rely on virus strain isolation and amplification, which present a challenge when dealing with highly pathogenic influenza viruses because many institutes or laboratories lack strict biological safety facilities. On the other hand, virus reassortment presents ethical issues because artificial viruses could cause a fatal pandemic. Therefore, a safe and convenient substitute model system is a precondition for active defense against influenza virus pandemics.

Pseudotyped viral particles (pp) are used widely as a reliable substitute for viruses that are difficult to isolate and culture in molecular biological studies of viral envelope proteins, viral receptor exploration, and neutralizing antibody (nAb) assays. Such studies have demonstrated the availability and utility of pp. For a long time, virologists ignored this technology because influenza viruses are easy to obtain. However, pseudotyped influenza viral particles offer the following advantages: there is no restriction due to biological safety, and reassortment based on hemagglutinin (HA) and neuraminidase (NA) is easy and without ethical issues. Thus, pseudotyped influenza viral particles combined with nAb assays might be optimized to create a surveillance system for assessing pandemic trends and the related disease burden.

In this study, a series of comparative analyses between live viruses and related pp were performed using the hemagglutination inhibition test and ELISA-, CPE-, and fluorescence-based microneutralization assays. Our findings provided a convenient and sensitive approach to the accurate assessment of virus-specific nAbs, which would improve pandemic influenza control and prevention.

## Materials and Methods

### Ethics Statement

This study was conducted according to the principles of the Declaration of Helsinki. The study was approved by the Ethics Committee of Institute of Medical Biology, Chinese Academy of Medical Sciences and Peking Union Medical College, permit number: [2013]1. All donors gave written informed consent for research use of blood samples. We also obtained written informed consent from the guardians on the behalf of the minors enrolled in our study (the ethics committee specifically approved the consent procedure for the participants between 16 and 18 years of age).

This study was carried out in strict accordance with the recommendations in the Guide for the Care and Use of Laboratory Animals of the National Institutes of Health. The animal study was also approved by the Committee on the Ethics of Animal Experiments of Institute of Medical Biology, Chinese Academy of Medical Sciences and Peking Union Medical College (Permit Number: [2013]3).

### Viruses

The HPAI virus, A/Anhui/1/2005 (H5N1), belonging to the 2.3.4 clade, was originally isolated from a fatal Chinese case [16]. The H5N1delta attenuated virus was provided by the US Centers for Disease Control (Atlanta, GA, USA); attenuation was achieved by deleting multiple cleavage sites in the HA. NYMC X-179A, influenza A(H1N1)PMD09 vaccine strain, influenza virus infectious NYMC X-179A, National Institute for Biological Standards and Control (NIBSC; London, UK) code 09/216, is a reassortant prepared by New York Medical College via the classical reassortant method from A/California/7/2009 (H1N1)v virus and NYMC X-157 virus, with the HA, NA, and PB1 genes donated from A/California/7/2009 (H1N1)v and the other internal genes donated from A/PR/8/34 (H1N1). The two viruses were propagated in chick embryos in a Biosafety Level II-enhanced facility.

### Pp production

cDNA fragments encoding the full-length HA of A/California/05/2009 (GenBank Acc. No. FJ966952) and the full-length NA of A/Ohio/07/2009 (GenBank Acc. No. FJ969534) were synthesized and inserted into an expression vector pCMV (Biovisualab, Shanghai, China) to produce constructs 09H1 and 09N1 as previously described [17]–[19], respectively. Similarly, cDNA fragments encoding the full-length HA and NA from H5N1 (A/Anhui/1/2005; GenBank Acc. No. DQ371928) were amplified and cloned into an expression vector pCMV to afford AH H5 and AH N1 expression systems. The capsid protein was from murine leukemia virus (MLV). The genomic RNA of the pseudovirus also carried a luciferase reporter gene.

The pp were produced by transfecting 293T cells with four plasmids: an HA expression plasmid pCMV-HA, an NA expression plasmid pCMV-NA, a gag-pol-encoding plasmid MLV-gag-pol, and a reporter plasmid CMV-GFP. HA and NA of naïve pp were derived from the same virus. For reassortant pp, HA and NA were from different viruses, including H5N1(09)pp, in which HA was AH H5 and NA was 09N1, and H1N1(H5)pp, in which HA was 09H1 and NA was AH N1. We carried out the following procedures: 1.5×10^6^ 293T cells cultured in a 9.6-cm dish were co-transfected with 0.8 µg of MLV-gag-pol, 0.8 µg of CMV-GFP, 0.3 µg of pCMV-HA and 0.3 µg of pCMV-NA by using Polyfect Transfection Reagent from QIAGEN (cat no. 301105; Hilden, Gernmany). After 6–8 h incubation, cells were washed once with phosphate-buffered saline and cultured in 3 mL complete Dulbecco minimum essential medium (Invitrogen, Carlsbad, CA, USA). At 48–72 h post-transfection, the pp were harvested from the supernatants of the transfected cells by filtration through a 0.45-µm Durapore PVDF membrane filter (Millipore Ireland, Cork, Ireland).

### Cell culture

MDCK, A549 targeting cells and 293T human embryo kidney pseudoparticle producer cells were obtained from the American Type Culture Collection (Manassas, VA, USA) and grown in Dulbecco's modified essential medium (Invitrogen, Carlsbad, CA, USA) supplemented with or without 10% fetal bovine serum.

### Hemagglutination and hemagglutination inhibition assay

The pp or virus samples were serially diluted 1∶2 in phosphate-buffered saline (PBS) in microplate wells (50 µL/well). Next, 50 µL aliquots of turkey red blood cells (1% suspension) were added to each well. Hemagglutination was scored 30 min later. Hemagglutination inhibition tests were performed according to standard methods [20]. To inactivate non-specific inhibitors, sera were treated with receptor destroying enzyme (RDE; Denka Seiken, Tokyo, Japan) overnight at 37°C and then inactivated by incubation at 56°C for 30 min. RDE-treated sera were serially diluted two-fold in v-bottom microtiter plates, starting at a 1∶10 dilution. An equal volume of virus or pp, adjusted to approximately 4 HA U/25 µL was added to each well. After a 30-min incubation at room temperature, 50 µL of 1% chicken erythrocytes were added to each mixture and incubated at room temperature after checking the RBC control for complete settling of the cells. The greatest serum dilution that displayed inhibition of hemagglutination was considered to represent the hemagglutination inhibition titer of the serum.

### ELISA-based microneutralization assay

Viruses or pp with 100 50% tissue culture infectious dose (TCID_50_)/well were incubated with two-fold serial dilutions of RDE-treated sera or NIBSC standard substance in a 96-well plate at 37°C in the presence or absence of TPCK-trypsin for 2 h. Then, 100 µL of freshly trypsinized MDCK cells (1.5×10^5^/mL) were added to each well (*i.e*., 1.5×10^4^ cells/well). After 18–22 h of incubation at 37°C and 5% CO_2_, the monolayers were washed with PBS and fixed in cold 80% acetone for 10 min at room temperature, then air-dried. The presence of viral protein was detected by ELISAs using a monoclonal antibody against the influenza A NP (clone F8) or HA, that is, NP for viruses and HA for pp. Anti-NP or -HA antibodies diluted 1∶4,000 in PBS containing 1% bovine serum albumin and 0.1% Tween 20 (E diluent) were added to each well and incubated at room temperature for 1 h. The plates were then washed and horseradish peroxidase-conjugated anti-mouse IgG (Kirkegaard and Perry Laboratories Inc., Gaithersburg, MD, USA) diluted 1∶2,000 in E diluent was added to each well, followed by further incubation for 1 h. After washing, *o*-phenylenediamine dihydrochloride was added and incubated at room temperature for 5 min. The reaction was stopped with the addition of an equal volume of 1 N sulfuric acid. The absorbance at 490 nm was then measured. The endpoint titer was expressed as the reciprocal of the highest dilution of serum with an OD value less than X, where X =  [(average of V+C wells) − (average of C wells)]/2+ (average of C wells) [21].

### CPE- or fluorescence-based microneutralization assays

Viruses or pp with 100 TCID_50_/well were incubated with two-fold serial dilutions of RDE-treated sera or NIBSC standard substance at 35°C (H1N1) or 37°C (H5N1 and pp) in the presence or absence of TPCK-trypsin for 1 h. The mixtures were then transferred to MDCK cell monolayers in a 96-well plate and incubated at 35 or 37°C for 1 h. After washing once, the cells were cultured in medium with TPCK-trypsin for virus or without for pp to prevent viral re-entry. The plates were then incubated at 35 or 37°C with 5% CO_2_ for 48–72 h. After this incubation period, the formation of a CPE or fluorescence was observed using a Nikon fluorescence microscope (Tokyo, Japan). Interpretation of the results was performed by comparing positive virus controls and negative cell controls. The nAb titer was defined as the highest dilution of serum or antibody at which 95% infectivity was blocked.

### Subjects and sera

In total, 270 serum samples were obtained from in- and out-patient subjects (age range, 16–56 years) in a cooperative hospital in Shanghai, China, in 2006. The subjects were considered to have not been exposed to A(H1N1)pmd09 influenza virus. An additional 286 samples were taken from another cooperative hospital in Kunming, China, in 2013. All study subjects were selected on the basis of a questionnaire designed to exclude any person with symptoms possibly associated with an influenza-like illness in the previous 6 months, or with an influenza virus vaccination history, especially the 2009 H1N1 vaccination for the 2013 subjects.

### NIBSC standard substance

NIBSC antiserum 07/338, Influenza antiserum A/Anhui/1/05 (H5), NIBSC code 07/338; NIBSC antiserum 07/150, International Standard for antibody to A/Vietnam/2004 H5N1 Clade 1 virus, NIBSC code 07/150, and NIBSC antiserum 09/152, influenza anti-A/California/7/2009 (H1N1)v HA serum, NIBSC code 09/152, were purchased from the NIBSC.

### Monoclonal antibodies and goat anti-influenza virus sera

Monoclonal antibodies were purchased from Biovisualab Inc. (Shanghai, China). Goat anti-influenza virus sera were raised by immunizing goats with purified H5N1delta or NYMC X-179A in our laboratory.

### Transmission electron microscopy

The supernatant of the medium containing pp was concentrated at 40,000 rpm for 25 min in a Hitachi centrifuge (Tokyo, Japan). Subsequently, both the original and concentrated supernatants were negative-stained, and the pp were observed by transmission electron microscopy (TECNAI 12; FEI, Blackwood, NJ) using an acceleration voltage of 80 kV.

### Statistical analysis

To estimate the value of the MN titer corresponding to the ELISA titer of anti-2009 and -2005 HA antibodies, we performed a correlation analysis using linear regression models. Data were analyzed using linear regression and multivariable models, Student's *t*-test, and estimation of geometric mean titers (GMTs) with confidence intervals and corresponding P-values, using SAS software (ver. 9.1). All data were expressed as the mean ± standard deviation or GMT (95% confidence interval [CI]).

## Results

### Reliability of the substitutability of viruses and pp in the hemagglutination inhibition test and ELISA-, CPE-, and fluorescence-based microneutralization assays

To evaluate the reliability of using pp as a substitute for the relevant influenza virus in nAb analysis, we first compared pp of A(H1N1)pmd09 (A/California/7/2009) and HPAI H5N1 (A/Anhui/1/2005) with their corresponding attenuated virus (NYMC X-179A and H5N1delta) in a hemagglutination inhibition test and ELISA-, CPE-, and fluorescence-based microneutralization assays. Goat antisera immunized with NYMC X-179A and H5N1delta and one anti-A(H1N1)pmd09 monoclonal nAb were used in these parallel tests. To ensure standardization and comparability, two NIBSC antisera against H5, 07/338 [A/Anhui/1/05 (H5)] and 07/150 [A/Vietnam/2004 H5N1 clade 1 virus], and one NIBSC antiserum against A/California/7/2009 (H1N1)v (09/152) were also included in these tests.

In the hemagglutination inhibition test, the viruses and pp were adjusted to 4 hemagglutination units according to WHO guidelines. As listed in Table 1, the highest dilution titers of NIBSC antiserum 07/338, NIBSC antiserum 07/150, and goat immune serum H5 to inhibit 4 hemagglutination units of H5N1delta and H5N1pp were (mean ± standard deviation) 613±46.19 and 533±184.75, 0.18±0.02 and 0.17±0.04 IU, and 5973±1478 and 4266±1478, respectively. The highest dilution titer of anti-H1 monoclonal antibody, NIBSC antiserum 09/152, and goat immune serum H1 to inhibit 4 hemagglutination units of NYMC X-179A and H1N1pp were 1.04±0.00 and 1.04±0.00 pmol, 1227±92.38 and 1280±0.00, and 2453±184.75 and 2560±0.00, respectively (Table 1, left).

**Table 1:** Comparative analysis of the viruses and pp by hemagglutination inhibition testing and ELISA-, CPE-, and fluorescence-based microneutralization assays.

	Hemagglutination inhibition^a^			ELISA-based Microneutralization^b^			CPE- and fluorescence-based microneutralization^b^		
	H5N1delta	H5N1pp	*P*	H5N1delta	H5N1pp	*P*	H5N1delta	H5N1pp	*P*
NIBSC antiserum 07/338	613±46.19^c^	533±184.75	0.535	1067±230.94	1000±346.41	0.795	853±184.75	960±0.00	0.423
NIBSC antiserum 07/150	0.18±0.02 IU	0.17±0.04 IU	0.749	0.13±0.02 IU	0.14±0.02 IU	0.519	0.16±0.03 IU	0.15±0.00 IU	0.423
Goat immune serum H5	5973±1478	4266±1478	0.230	4000±0.00	5000±1732.05	0.423	5333±1847.52	5867±923.76	0.678
	NYMC X-179A	H1N1pp	*P*	NYMC X-179A	H1N1pp	*P*	NYMC X-179A	H1N1pp	*P*
Anti-H1 monoclonal antibody	1.04±0.00 pmol	1.04±0.00 pmol	–	0.83±0.00 pmol	0.69±0.12 pmol	0.184	1.04±0.00 pmol	0.87±0.15 pmol	0.423
NIBSC antiserum 09/152	1227±92.38	1280±0.00	0.423	2400±0.00	2933±461.88	0.184	1707±369.50	1920±0.00	0.184
Goat immune serum H1	2453±184.75	2560±0.00	0.423	3000±0.00	4667±1154.7	0.130	3200±0.00	4266±923.76	0.184

a For the hemagglutination inhibition test, the titers of the virus and pp were adjusted to 4 hemagglutination units.

b For the microneutralization assays, the titers of the virus and pp were adjusted to 100 TCID50. The 95% inhibitory concentration (IC95) was used for nAb analysis.

c Titers are presented as the average ± standard deviation. All tests were repeated three times.

For the microneutralization assays, the viruses and pp were adjusted to 100 TCID_50_ according to WHO guidelines. The highest dilution titer of NIBSC antiserum 07/338, NIBSC antiserum 07/150, and goat immune serum H5 to neutralize 100 TCID_50_ H5N1delta and H5N1pp were 1067±230.94 and 1000±346.41, 0.13±0.02 and 0.14±0.02 IU, and 4000±0.00 and 5000±1732.05, respectively. The highest dilution titer of anti-H1 monoclonal antibody, NIBSC antiserum 09/152, and goat immune serum H1 to neutralize 100 TCID_50_ NYMC X-179A and H1N1pp were 0.83±0.00 and 0.69±0.12 pmol, 2400±0.00 and 2933±461.88, and 3000±0.00 and 4667±1154.7, respectively (Table 1, middle). In conclusion, the H5N1pp displayed very similar characteristics to H5N1delta in the microneutralization assay. H1N1pp displayed slightly lower sensitivity to the anti-H1 monoclonal antibody and slightly greater sensitivity to NIBSC antiserum 09/152 and goat immune serum H1.

In the CPE- and fluorescence-based microneutralization assays, the viruses and pp were first adjusted to 100 TCID_50_ according to WHO guidelines. The highest dilution titer of NIBSC antiserum 07/338, NIBSC antiserum 07/150, and goat immune serum H5 to inhibit the CPEs caused by 100 TCID_50_ H5N1delta and H5N1pp were 853±184.75 and 960±0.00, 0.16±0.03 and 0.15±0.00 IU, and 5333±1847.52 and 5867±923.76, respectively. The highest dilution titer of the anti-H1 monoclonal antibody, NIBSC antiserum 09/152, and goat immune serum H1 to inhibit the CPEs caused by 100 TCID_50_ NYMC X-179A and H1N1pp were 1.04±0.00 and 0.87±0.15 pmol, 1707±369.50 and 1920±0.00, and 3200±0.00 and 4266±923.76, respectively (Table 1, right). In conclusion, the H5N1pp and H1N1pp displayed very similar characteristics to their relative viruses in CPE- and fluorescence-based microneutralization assays.

### Comparative analysis of the viruses and pp in influenza virus-specific nAb detection using population-based sera

The above experiments demonstrated the substitutability of the viruses and pp in the hemagglutination inhibition test and in ELISA-, CPE-, and fluorescence-based microneutralization assays. To further evaluate the reliability of using pp as a substitute for relevant influenza viruses in nAb analysis, we compared the characteristics of the pp with their corresponding viruses in population sera detection.

The first set of sera, collected from 286 subjects in 2013, represented the population immunity characteristics following the A(H1N1)pdm09 pandemic. Comparative parallel tests were performed using these sera first.

NYMC X-179A and the H1N1pp detected a similar number of subjects with nAbs against A(H1N1)pmd09. The titers against NYMC X-179A and H1N1pp as measured by a hemagglutination inhibition test and by ELISA-, CPE-, and fluorescence-based microneutralization assays were (GMT [95% CI]) 25.78 (22.09, 30.08) and 34.79 (29.95, 40.41), 24.67 (20.96, 29.05) and 24.17 (20.62, 28.33), and 19.48 (16.79, 22.59) and 21.58 (18.56, 25.10), respectively (Table 2).

**Table 2:** Comparative analysis of the viruses and pp in influenza virus-specific nAb detection using population-based sera.

	Hemagglutination inhibition^a^		ELISA-based Microneutralization^b^		CPE- and fluorescence-based microneutralization^b^	
	H5N1delta	H5N1pp	H5N1delta	H5N1pp	H5N1delta	H5N1pp
286 samples collected in 2013						
Positive rate	6/286	7/286	6/286	6/286	4/286	6/286
GMT (95%CI)	17.82 (8.72, 36.43)	14.86 (8.97, 24.61)	12.01 (8.79, 16.42)	10.7 (9.00, 12.73)	11.07 (8.01, 15.28)	10.7 (9.00, 12.73)
*P*	0.604		0.426		0.779	
R^2^	0.924		1.000		0.814	
270 samples collected in 2006						
Positive rate	5/270	5/270	5/270	5/270	5/270	5/270
GMT (95%CI)	20.41 (8.14, 63.55)	19.36 (7.89, 61.35)	21.10 (8.32, 118.02)	17.68 (8.01, 128.00)	17.83 (7.81, 59.44)	19.14 (8.91, 118.25)
*P*	0.658		0.754		0.385	
R^2^	1.000		1.000		1.000	
	NYMC X-179A	H1N1pp	NYMC X-179A	H1N1pp	NYMC X-179A	H1N1pp
286 samples collected in 2013						
Positive rate	153/286	149/286	144/286	140/286	136/286	141/286
GMT (95% CI)	25.78 (22.09, 30.08)	34.79 (29.95, 40.41)	24.67 (20.96, 29.05)	24.17 (20.62, 28.33)	19.48 (16.79, 22.59)	21.58 (18.56, 25.10)
*P*	0.006		0.858		0.338	
R^2^	0.972		0.972		0.966	
270 samples collected in 2006						
Positive rate	68/270	68/270	68/270	68/270	68/270	68/270
GMT (95% CI)	33.54 (7.56, 179.01)	30.54 (7.86, 234.41)	63.45 (17.45, 324.45)	63.58 (17.45, 213.12)	56.42 (19.35,214.12)	59.32 (15.56,236.58)
*P*	0.724		0.625		0.515	
R^2^	1.000		1.000		1.000	

a For the hemagglutination inhibition test, the titers of the virus and pp were adjusted to 4 hemagglutination units.

b For the microneutralization assays, the titers of the virus and pp were adjusted to 100 TCID50. The 95% inhibitory concentration (IC95) was used for nAb analysis.

In comparison, H5N1delta and the H5N1pp detected a similar number of subjects with nAbs against H5N1. The titers against H5N1delta and H5N1pp as detected by a hemagglutination inhibition test and by ELISA-, CPE-, and fluorescence-based microneutralization assays were 17.82 (8.72, 36.43) and 14.86 (8.97, 24.61), 12.01 (8.79, 16.42) and 10.7 (9.00, 12.73), and 11.07 (8.01, 15.28) and 10.7 (9.00, 12.73), respectively (Table 2).

In conclusion, by qualitative detection, the pseudotyped particles showed complete agreement with the corresponding virus, with an R^2^ value equal to or near 1. By quantitative detection, although the H1N1pp were more sensitive than NYMC X-179A, the pseudotyped particles showed fairly high consistency with the corresponding virus.

The second set of sera, collected from 270 subjects in 2006, represented the population immunity characteristics before the A(H1N1)pdm09 pandemic. To further evaluate the reliability of using pp in nAb detection, comparative parallel tests were performed using these sera.

NYMC X-179A and the H1N1pp detected the same 68 of 270 serum samples carrying nAbs against A(H1N1)pdm09. The titers against NYMC X-179A and the H1N1pp as detected by a hemagglutination inhibition test and by ELISA-, CPE-, and fluorescence-based microneutralization assays were 33.54 (7.56, 179.01) and 30.54 (7.86, 234.41), 63.45 (17.45, 324.45) and 63.58 (17.45, 213.12), and 56.42 (19.35,214.12) and 59.32 (15.56,236.58), respectively (Table 2).

In comparison, H5N1delta and the H5N1pp detected the same 5 of 270 serum samples with nAbs against H5N1. The titers against H5N1delta and the H5N1pp as detected by a hemagglutination inhibition test and by ELISA-, CPE-, and fluorescence-based microneutralization assays were 20.41 (8.14, 63.55) and 19.36 (7.89, 61.35), 21.10 (8.32, 118.02) and 17.68 (8.01, 128.00), and 17.83 (7.81, 59.44) and 19.14 (8.91, 118.25), respectively (Table 2).

In conclusion, using both qualitative and quantitative detection methods, the pseudotyped particles showed fairly high consistency with the corresponding virus. Furthermore, the immune protection level of the population against A(H1N1)pdm09 was high.

### Establishment of an influenza virus pandemic surveillance system

The influenza virus pandemic surveillance system described in this report included two major elements: 1) predictions of HA and NA matching with reassortant pp, and 2) evaluation of the background protection level of population immunity against a new reassortant using valid reassortant pp.

Toward realizing this goal, we produced reassortant pp, including H5N1(09)pp and H1N1(H5)pp. To study the population immunity protection level, the above two reassortant pp as well as naïve pp (H1N1pp and H5N1pp) were used to perform hemagglutination inhibition testing and CPE- and fluorescence-based microneutralization assays on the second group of serum samples.

The H5N1pp and H5N1(09)pp detected the same 5 of 270 serum samples containing nAbs against H5. The anti-H5N1 and -H5N1(09)pp titers (GMT [95% CI]) in the hemagglutination inhibition test and CPE- and fluorescence-based microneutralization assay were 19.36 (7.89, 61.35) and 18.35 (8.01, 55.38), and 19.14 (8.91, 118.25) and 16.57 (8.11, 109.63), respectively (Table 3).

**Table 3:** Assessment of population nAb detection using reassortant pp.

	Hemagglutination inhibition^a^		CPE- and fluorescence-based microneutralization^b^	
	H5N1pp	H5N1(09)pp	H5N1pp	H5N1(09)pp
270 samples collected in 2006				
Positive rate	5/270	5/270	5/270	5/270
GMT (95% CI)	19.36 (7.89, 61.35)	18.35 (8.01, 55.38)	19.14 (8.91, 118.25)	16.57 (8.11, 109.63)
*P*	0.698		0.657	
R^2^	1.00		1.00	
	H1N1pp	H1N1(H5)pp	H1N1pp	H1N1(H5)pp
270 samples collected in 2006				
Positive rate	68/270	68/270	68/270	68/270
GMT (95% CI)	30.54 (7.86, 234.41)	41.35 (8.96, 214.58)	59.32 (15.56, 236.58)	58.54 (18.32, 214.65)
*P*	0.678		0.576	
R^2^	1.00		1.00	

a For the hemagglutination inhibition test, the titers of the virus and pp were adjusted to 4 hemagglutination units.

b For the microneutralization assays, the titers of the virus and pp were adjusted to 100 TCID50. The 95% inhibitory concentration (IC95) was used for nAb analysis.

In comparison, the H1N1pp and H1N1(H5)pp detected the same 68 of 270 serum samples with nAbs against H1. The anti-H1N1pp and -H1N1(H5)pp titers in the hemagglutination inhibition test and CPE-based microneutralization assay were 30.54 (7.86, 234.41) and 41.35 (8.96, 214.58), and 59.32 (15.56, 236.58) and 58.54 (18.32, 214.65), respectively (Table 3).

In summary, both the naïve and reassortant pp displayed complete agreement with each other using qualitative detection methods (R^2^ = 1). The average nAb titers against reassortant pp were slightly lower than that against the native pp. These findings suggested that pseudotyped influenza viral particles combined with an nAb assay might be optimized to constitute a surveillance system for pandemic trends and the related disease burden.

### Comparative analysis of the convenience of using viruses and pp

To evaluate the convenience of using viruses versus pp for antibody detection, we compared them in terms of biosafety regulation, ethical issues, virus isolation, amplification and purification, virus titration, and antibody assays using three methods (Table 4). For the viruses, the biosafety regulation was Level III, ethical issues were involved, and virus isolation, amplification, and purification were required and time-consuming. For the pp, the biosafety regulation was Level II, ethical issues were not involved, and the time required for pp production was comparatively shorter. The time required to perform the microneutralization assay based on the observation of CPEs using virus was about 3–4 days, while that of the microneutralization assay based on the observation of fluorescence using pp was about 2–3 days.

**Table 4:** Comparison of live virus versus pp.

	Hemagglutination inhibition		ELISA-based Microneutralization		CPE- and fluorescence-based microneutralization	
	Live virus	pp	Live virus	pp	Live virus	pp
Principle	Antibodies can block the ability of viral particles to agglutinate red blood cells and to bind to N-acetylneuraminic acid.		nAbs can be detected based on the reduction in virus-infected cells in combination with ELISA using microtiter plates.		nAbs can be detected based on the reduction in virus-infected cells using the observation of CPEs or fluorescence.	
Biosafety regulation	Level III^c^	Level II	Level III	Level II	Level III	Level II
Ethical issues	Yes	No	Yes	No	Yes	No
Virus isolation, amplification, and purification	Yes	No	Yes	No	Yes	No
Sampling	1 d	-	1 d	-	1 d	-
Cell or chick embryo based virus isolation	3–7 d	-	3–7 d	-	3–7 d	-
Virus titration^a^	1 h	1 h	3–4 d	2–3 d	3–4 d	2–3 d
Collection and treatment of sera	1 d	1 d	1 d	1 d	1 d	1 d
Antibody assay	2 hrs	2 hrs	1 d	1 d	3 d	2–3 d
Viral replication^b^	-	-	≧2	-	≧6	-
Viral genome cloning	No	3 d	No	3 d	No	3 d
Pp production	-	2–3 d	-	2–3 d	-	2–3 d
Total time consumption	5–9 d	6–7 d	9–14 d	9–11 d	11–16 d	10–13 d

a For the hemagglutination inhibition test, titration refers to the hemagglutination test. For the ELISA-, CPE-, and fluorescence-based microneutralization assays, titration refers to the TCID.

b refers to the possible replication number.

c refers to highly pathogenic influenza virus.

### The use of pp in nAb detection and electron microscopic observation

**Figure 1:**
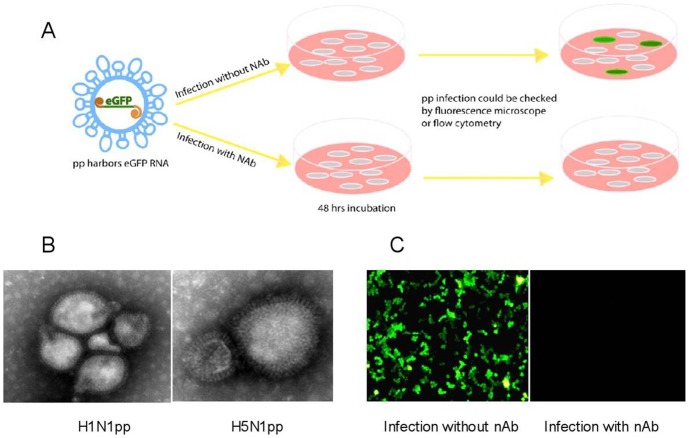
Use of pp in nAb detection and electron microscopic observation. A. The pp were composed primarily of three viral elements: gag-pol, the viral mimic genome, and a reporter gene (*e.g*., eGFP) were incorporated into the artificial viral genome. B. Transmission electron microscopic image showing H5N1pp and H1N1pp. C. The pp-infected cells expressed GFP and could be visualized by fluorescence microscopy; nAbs were quantified based on the GFP: cell ratio.

The schematic diagram in Fig. 1 was provided to help explain the principle of using pp in nAb detection. The pp were composed primarily of three viral elements: gag-pol, which forms the viral structure, recognizes the viral genome, and is responsible for the genome lifecycle; the viral mimic genome, which provides the genomic elements that will be recognized by gag-pol and ensures complete viral RNA metabolism (in addition, a reporter gene [*e.g*., eGFP] was incorporated into the artificial viral genome); and envelope proteins, which are presented on the artificial viral particle (Fig. 1A). Thus, the pp-infected cells expressed GFP and could be visualized by fluorescence microscopy within 2–3 days (Fig. 1C).

To verify the pp, we observed them using transmission electron microscopy. The pp were visible as particles surrounded by HA and NA spikes, in accordance with the typical morphology of influenza virus (Fig. 1B). Meanwhile, no typical viral morphology of influenza virus was found in either the supernatant from naïve 293T cells or in the supernatant from 297T cells transfected with a gag-pol-encoding plasmid and eGFP reporter plasmid but no HA or NA expression plasmids.

## Discussion

This study presented the first evaluation of the reliability of using pseudotyped influenza viral particles in nAb detection. The reliability was shown in several ways. First, hemagglutination inhibition tests, as well as ELISA-, CPE-, and fluorescence-based microneutralization assays, demonstrated that the pp performed much the same as related viruses in terms of nAb detection. Second, the reliability of using pp was confirmed using the vaccine strains for A(H1N1)pmd09 (A/California/7/2009) and HPAI H5N1 (A/Anhui/1/2005). Third, the reliability was verified using two population groups, representing the population immunity situation before and after the A(H1N1)pmd09 outbreak; the nAb-positive rate of the latter was much higher than that of the former. After we demonstrated the reliability of using pseudotyped influenza viral particles in nAb detection, we generated reassortant pp using the NA and HA of A(H1N1)pmd09 and HPAI H5N1 (A/Anhui/1/2005) to examine the possibility of pp in emerging influenza disease burden estimation. For pp harboring either H5 [H5N1pp and H5N1(09)pp] or H1 [H1N1pp and H1N1(H5)pp], strong consistency was detected in both quantitative and qualitative nAb analyses.

The host immune barrier might be caused by cross-infection or an inapparent infection, and all pathogens have their own evolutionary process. For example, despite the initial concern that little protective immunity against the novel H7N9 virus existed in the general population, subsequent epidemiological studies suggested the occurrence of pre-existing immunity [22]. Recently, cross-reactive antibodies to this novel H7N9 influenza virus were demonstrated to be induced by immunization with a North American lineage H7 subtype HA [23]. According to a recent report, antibodies against the HA of the novel H7N9 virus were present in serum samples from the general population in southern Vietnam [24]. Thus, the investigation of “pre-existing” immunity against a certain emerging influenza virus will obviously improve its control and prevention scientifically and objectively.

Both population groups displayed very low nAb-positive rates against H5N1 virus and pp harboring H5, suggesting that the immunity level to this virus was very low. Of the nAbs against A(H1N1)pmd09 and pp harboring the same HA, the rate of positive sera sampled in 2006 was less than that of the sera sampled in 2013. This suggested that after the A(H1N1)pmd09 pandemic, populations were immunized broadly to a certain extent. The nAb titers of the sera sampled in 2006 were slightly higher than those of the sera sampled in 2013. This might be due to the difference in population. The sera sampled in 2006 demonstrated high pre-existing immunity to the A(H1N1)pmd09, which was consistent with a strong pre-existing immunity against A(H1N1)pmd09 reported in Shanghai [8]. The Centers for Disease Control and Prevention (Atlanta, GA, USA) also reported that among persons >60 years old, 34% had pre-existing, cross-reactive neutralizing antibodies against A(H1N1)pmd09 [25]. Many previous studies performed on geographically diverse populations have demonstrated pre-existing neutralizing antibodies [11], [12], [25]–[30]. Many of them suggested that pre-existing immunity in elderly subjects had been elicited by infection with 1918-related H1N1 virus [12], [25], [27]–[30]. However, in mainland China, due to the extremely high human, swine, and poultry population density, and environmental contamination, influenza viruses might cause undetected infections. Another reason for pre-existing immunity may be that many subjects had been exposed repeatedly to unidentified viruses, including the virus strains with hemagglutinins related with A(H1N1)pmd09 antigenically [8].

Virological and molecular virological characterization of HA and NA expression and maturation, HA and NA incorporation into pp, the conformational and functional consistency of HA and NA between pp and the corresponding wild-type virus, pp stability and infectivity, and so on have been well characterized in our and other researchers' studies [31]–[35]. However, the reliability of using pseudotyped influenza viral particles in nAb detection has not been described previously. As the gold standard for viral nAb assessment, in our CPE-based microneutralization assay the use of pp was much more convenient than the use of virus, although the CPEs were indistinguishable. The results of pp after 48 hours were much the same as that after 72 hours, while the CPE was more clear in virus tests. This was maybe because pp could not replicate, whereas viruses could replicate many times.

The pp were also applied for other novel influenza viruses, and a recent study developed pseudotyped viral particles to detect neutralizing antibodies against H7N9 [35]. Moreover, reassortment of the two major antigens (HA and NA) was easy to achieve using pp and did not carry ethical restrictions. Therefore, the use of pseudotyped particles offers many advantages when compared to the use of live virus in a surveillance system for analyzing pandemic trends and the related disease burden.

The pp were a single cycle system and not able to capture events downstream of entry/fusion such as inhibition of egress and inhibition of HA maturation. However, these downstream inhibition steps have been described for many broadly neutralizing monoclonal antibodies and they might be important for heterosubtypic immunity. Therefore, there is a limitation of pp in that the pp system cannot represent these biological processes of corresponding virus.
